# Planktonic foraminifera genomic variations reflect paleoceanographic changes in the Arctic: evidence from sedimentary ancient DNA

**DOI:** 10.1038/s41598-020-72146-9

**Published:** 2020-09-15

**Authors:** Joanna Pawłowska, Jutta E. Wollenburg, Marek Zajączkowski, Jan Pawlowski

**Affiliations:** 1 Institute of Oceanology Polish Academy of Sciences, Sopot, Poland; 2 Alfred Wegener Institute, Bremerhaven, Germany; 3 University of Geneva, Geneva, Switzerland

**Keywords:** Ecology, Environmental sciences, Ocean sciences, Palaeoceanography, Palaeoclimate, Eukaryote, Metagenomics

## Abstract

Deciphering the evolution of marine plankton is typically based on the study of microfossil groups. Cryptic speciation is common in these groups, and large intragenomic variations occur in ribosomal RNA genes of many morphospecies. In this study, we correlated the distribution of ribosomal amplicon sequence variants (ASVs) with paleoceanographic changes by analyzing the high-throughput sequence data assigned to *Neogloboquadrina pachyderma* in a 140,000-year-old sediment core from the Arctic Ocean. The sedimentary ancient DNA demonstrated the occurrence of various *N. pachyderma* ASVs whose occurrence and dominance varied through time. Most remarkable was the striking appearance of ASV18, which was nearly absent in older sediments but became dominant during the last glacial maximum and continues to persist today. Although the molecular ecology of planktonic foraminifera is still poorly known, the analysis of their intragenomic variations through time has the potential to provide new insight into the evolution of marine biodiversity and may lead to the development of new and important paleoceanographic proxies.

## Introduction

The diversity, biology and shell chemistry of planktonic foraminifera are extremely sensitive to physical–chemical changes on the ocean surface. Aside from being one of the major components of sea-floor sediments^1^, planktonic foraminifera are one of the key groups of microfossils used in paleoclimatic and paleoenvironmental research^2–4^. Planktonic foraminifera are composed of approximately 50 morphospecies living in the modern oceans^5^. Genetic studies have shown that nearly every morphospecies is composed of several different genotypes^6,7^, which modern distribution may reflect different ecological conditions^8–10^. The population of *N. pachyderma* in the Atlantic Ocean and polar regions consists of a complex of seven distinct genotypes with different biogeographic distribution^6,7^. *N. pachyderma* Type I diverged between 1.8 and 1.5 Ma from the other type^8^ and it’s the only type inhabiting northern high latitudes^7^. Moreover, some genomic variations are also observed within the different genotypes or even within single cells^11^. The later phenomenon, named intragenomic polymorphism, has been extensively studied in benthic foraminifera^12^ and also observed in planktonic species^11^.

Recent advancements in meta-genomic techniques allows for simultaneous identification of many taxa in environmental DNA (eDNA) samples via high-throughput amplicon sequencing (so called metabarcoding). Recent metabarcoding studies from water and surface sediments have confirmed the large genetic diversity of planktonic foraminifera, which significantly exceeds the number of their morphotypes^13,14^. This is particularly evident in small-sized foraminifera, whose ecological importance is often underestimated^14^. It has been shown that the distribution of planktonic foraminifera DNA in surface sediments is reflective of community structure^15^. However, until now, no metabarcoding studies have analyzed the composition of planktonic foraminifera assemblages in sediment ancient DNA (*seda*DNA) samples.

Numerous studies have reported the preservation of DNA in marine sediments over tens to a hundred thousand years^16–18^, but longer preservations, up to 1.4 Ma, also appear to be possible^19^. Recently, *seda*DNA metabarcoding studies have been increasingly used to track past climatologic and environmental changes, e.g., for tracing Holocene environmental changes in bacterial microbiomes^20,21^, planktonic microbial eukaryote communities^22,23^, and Arctic benthic foraminifera^24,25^. *Seda*DNA metabarcoding analyses have also been applied to deep-sea sediments of the South Atlantic dating back to 35 ka, which revealed a high diversity of foraminifera and radiolarians^18^. However, this is the first study to investigate the composition of Arctic planktonic foraminifera in *seda*DNA samples.

We focused on *Neogloboquadrina pachyderma*, the dominant planktonic foraminifera species in high latitudes^26,27^, and one of the most important tools for reconstructing past climatic and ocean surface conditions in the North Atlantic and Arctic Oceans^28–30^. The species shows morphological variability, displaying at least 5 different morphotypes^31^, and a complex of 7 distinct genetic types^6,8,32^ has been described for the global population^6,32^. However, the high northern latitudes are inhabited exclusively by *N. pachyderma* Type I^6,32,33^, for which two ribosomal sequences are publicly available^11^.

Here we present the first results of an investigation into the genomic variability of *N. pachyderma* Type I that has been inferred from *seda*DNA in Arctic Ocean sediments spanning the last 140,000 years. It is, to our knowledge, the oldest known foraminiferal *seda*DNA record. Our study reports the changes of relative abundance of *N. pachyderma* Type I genomic variants through time. It demonstrates the potential of *seda*DNA metabarcoding as a source of new paleoceanographic proxies and opens new avenues for expanding the use of ancient DNA in paleoceanographic research.

## Results

**Figure 1:**
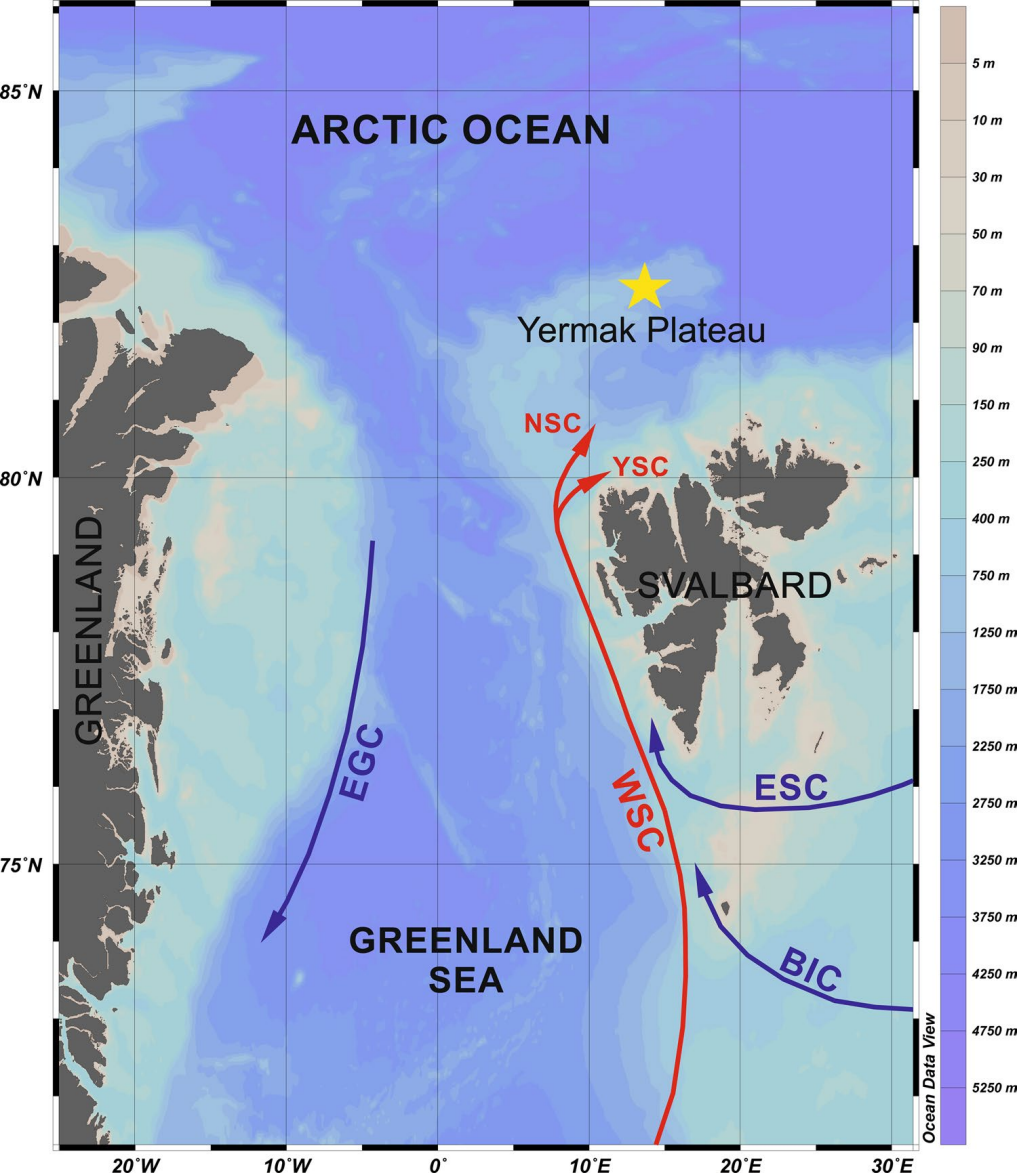
Oceanographic setting of the study area. Map was generated using Ocean Data View 5.3.0 (Schlitzer, Reiner, Ocean Data View, odv.awi.de, 2020). Coring station is marked by yellow star. Major sea currents are marked by arrows. WSC—West Spitsbergen Current, ESC—East Spitsbergen Current, BIC—Bear Island Current, NSC—North Spitsbergen Current, YSC—Yermak Slope Current, EGC—East Greenland Current.

In 2015 core PS92/0039–2 was collected from 1,464 m water depth on the eastern flank of the Yermak Plateau (Eurasian Basin, Arctic Ocean) (Fig. 1). The presented results originate from sequencing of sediment samples taken at 5 cm intervals from this 850 cm long core, yielding a total of 170 analyzed samples. Sequences of *N. pachyderma* were recorded in 78 out of 170 analyzed sediment samples. Among these, 48 samples contained more than 100 N*. pachyderma* sequences.

High-throughput sequencing yielded 3,550,037 sequence reads obtained for an 80-base-pair-long fragment of the 37f region of foraminiferal 18S rRNA gene. Sequences were clustered into 42,329 Amplicon Sequence Variants (ASVs) with 143 of them containing more than 1,000 reads (Table 1). The number of reads in each sample in presented on Supplementary Fig. 1

**Table 1:** The total number of ASVs and number of reads found in the dataset.

	No of ASVs	No of reads
Total number	42, 329	3,550,037
> 1,000 reads	143	3,243,932

### Characterization of N. pachyderma ASVs

Among the ASVs containing more than 1,000 sequence reads, 12 ASVs represented by 507,466 reads, were assigned to *N. pachyderma* Type I (Table 2). Three ASVs (5, 7 and 10), dominated the *N. pachyderma* Type I dataset with 75% of reads. The proportion of five ASVs (16, 18, 38, 47, and 60) ranged from 1 to 10%, while three ASVs (57, 79, 88) were represented by less than 1% (Table 2).

**Table 2:** Number of reads and percentage of sequences clustered into ASVs assigned to *Neogloboquadrina pachyderma,* and number of samples in which each ASV occurred.

ASV number	No of reads	Percentage	No of samples
ASV 5	168 874	33%	35
ASV 7	144 567	28%	19
ASV 10	74 432	14%	34
ASV 16	42 417	8%	11
ASV 18	38 088	7%	20
ASV 38	13 994	2.75%	7
ASV 47	7 998	1.5%	8
ASV 60	5 554	1%	18
ASV 57	4 360	< 1%	11
ASV 79	3 243	< 1%	8
ASV 88	2 517	< 1%	2
ASV 117	1 422	< 1%	6

**Figure 2:**
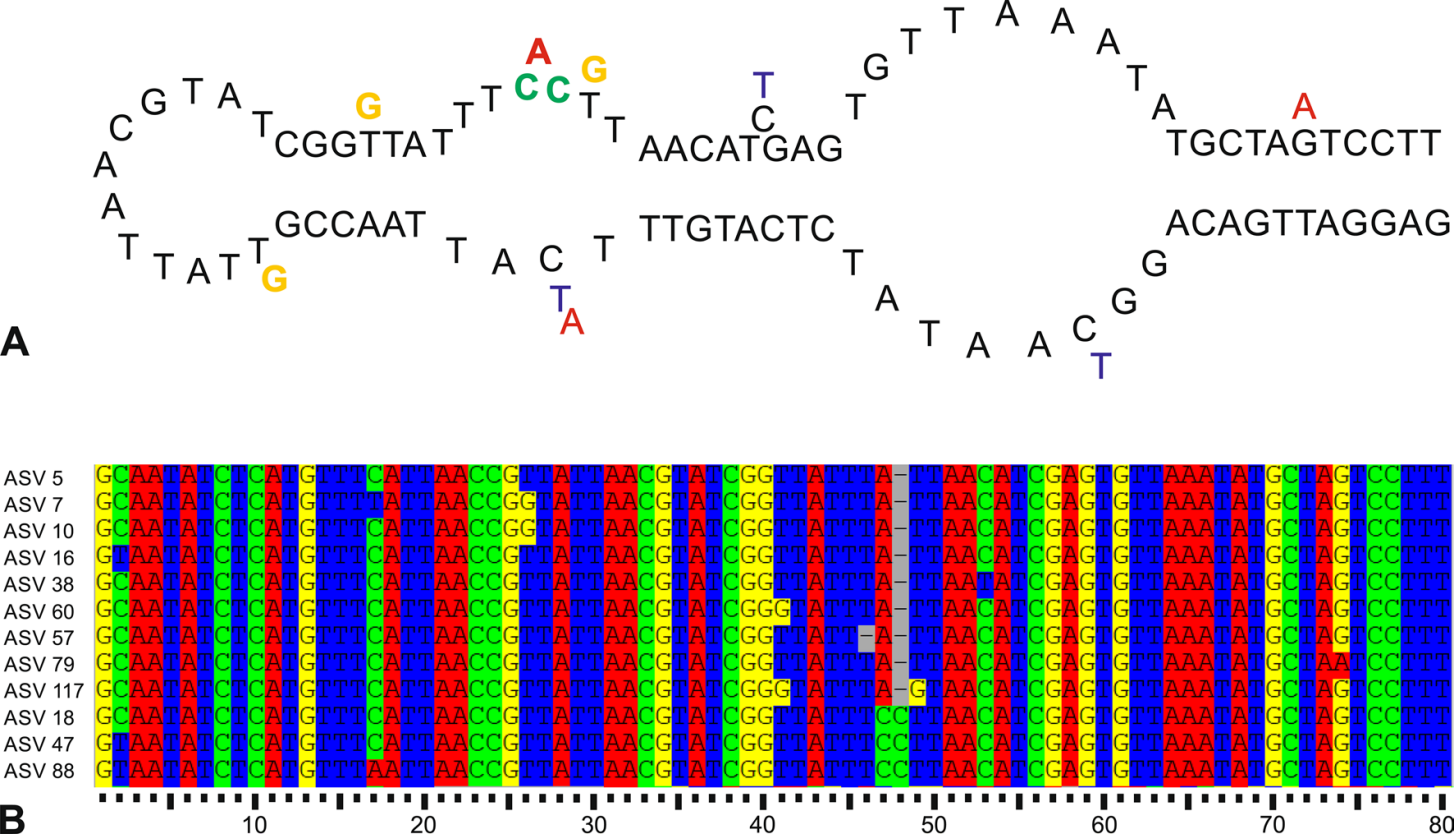
Secondary DNA structure of the 37f. hypervariable region of *N. pachyderma* Type I (**A**) and alignments represents *N. pachyderma* ASVs found in the studied core (**B**). Homologous nucleotides are presented in successive columns and each nucleotide is marked with representative color: A—red, C—green, G—yellow, T—blue. The position of each nucleotide is marked with scale.

The majority of variations between ASVs in the 80-base-pair-long fragment were single-nucleotide substitutions, which were recorded at 8 positions (Fig. 2). Substitutions, which are replacements of a nucleotide by another (marked by letters A, C, T, G), are the most common DNA mutations. Depending on which nucleotides are being replaced, this mutation may be called transition or transversion. Among the 15 substitutions, there were 5 C-T transitions, 1 A-G transition, 5 G-T transversions, and 4 C-A transversions. In addition, an insertion of C was detected at position 48. This insertion was followed by the substitution of A with another C. This change of a single A replaced by a double C was observed in the abundantly sequenced ASV 18, and in the more rare ASVs 47 and 88 (Fig. 2).

### Variations of N. pachyderma genotypes over the last 140 cal ka BP

**Figure 3:**
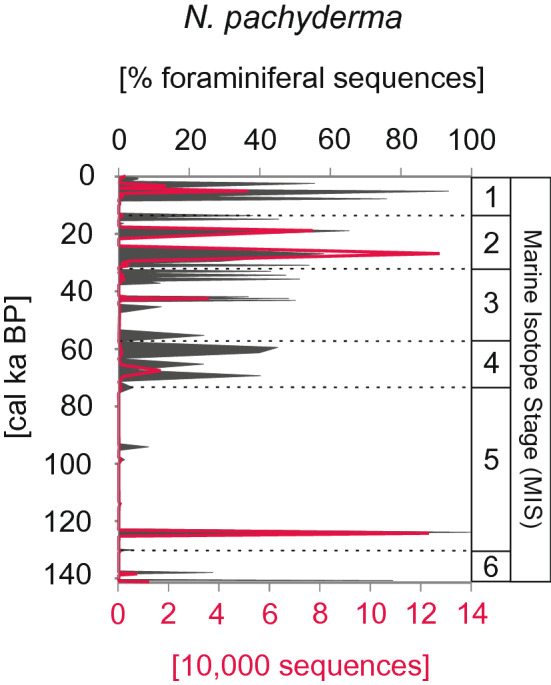
The relative abundance of *N. pachyderma* sequences in the studied core expressed as percentage of foraminiferal sequences (grey shading) and number of *N. pachyderma* sequence reads (red line). The presented data includes all ASVs.

In the oldest part of the core (Marine Isotope Stage (MIS) 6), only single peaks of *N. pachyderma* sequences were recorded at 138 and 130 cal ka BP. The interval between 130 and 71 cal ka BP (i.e., MIS 5) was nearly barren of *N. pachyderma* DNA, with only minor peaks of sequences noted at 115, 98 and 94 cal ka BP. The *N. pachyderma* sequences were recorded continuously during MIS 4 (prior to ~ 58 cal ka BP) and accounted for up to 45% of the foraminiferal (both benthic and planktonic) sequences. During MIS 3, *N. pachyderma* sequences were absent in sediment layers dated to ~ 50 and 40 cal ka BP. In the other periods, *N. pachyderma* constituted ~ 40% of the foraminiferal sequences. Both MIS 2 and MIS 1 (29 cal ka BP to the present) were marked by the highest percentages of *N. pachyderma*, which constituted up to 94% of the foraminiferal sequences at 5 cal ka BP (Fig. 3).

**Figure 4:**
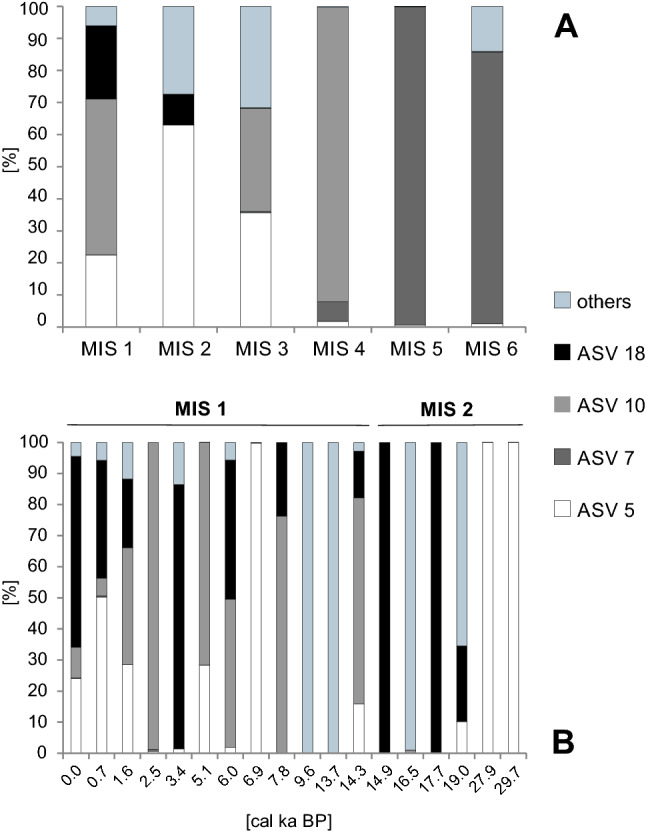
The relative abundance of the ASVs (expressed as % of *N. pachyderma* sequences) found in the samples referring to each MIS (**A**) and the occurrence of ASVs in each sample referring to MIS 1 and MIS 2 (**B**).

**Figure 5:**
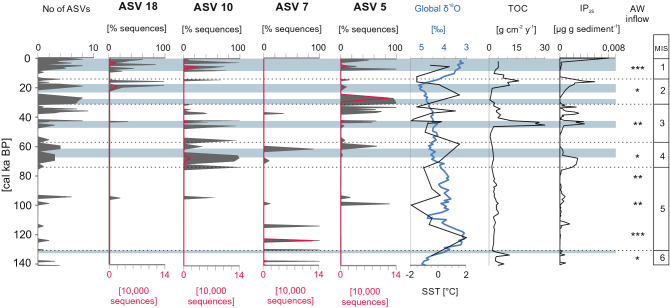
The number of ASVs in each sample, the relative abundance of the dominant ASVs found in the studied core, expressed as percentage of *N. pachyderma* sequences (grey shading) and number of sequence reads (red line). The global δ^18^O (blue line) is presented after^66^. OH-GDGT-based SSTs were calculated according to the RI-OH’ index recommended for polar regions^67^. The accumulation rate of organic carbon (TOC) and IP_25_ are presented after^45^. The advection of AW to the Arctic is marked by stars, from less intensive (*) to the most intensive (***). Periods with less severe ice cover are marked with light blue shading. AW inflow and sea-ice data are presented after^45^.

The occurrence and percentages of different ASVs varied through time (Figs. 4, 5). Figure 5 shows changes in the genetic composition of *N. pachyderma* during each MIS (Fig. 4a) and within MIS’ 1 and 2 (Fig. 4b). Figure 5 illustrates the variations for each ASV separately.

During MIS 6 (prior to ~ 130 cal ka BP), only single peaks of ASV 7 and ASV 79 were recorded at 138 and 130 cal ka BP. These ASVs represented up to 100% and 20% of *N. pachyderma* sequences, respectively (Figs. 4, 5 and Supplementary Fig. 2).

During MIS 5 (~ 130–71 cal ka BP), ASV 7 made up the majority of *N. pachyderma* sequences (Fig. 4). This ASV was recorded at 124, 114 and 99 cal ka BP. Single peaks of ASVs 5 and 10 were also recorded at 99 and 94 cal ka BP, respectively (Fig. 5). Moreover, ASVs 18 and 88 occurred at 95 cal ka BP, representing 20% and 7.9% of the *N. pachyderma* sequences, respectively (Fig. 5).

From MIS 4 71 cal ka BP) to the present, the *N. pachyderma* assemblage shifted from being dominated by a single ASV 10 to a co-dominance of several ASVs (10, 5, 7, and 57). ASV 10 dominated the *N. pachyderma* assemblage in early MIS 4 (prior to ~ 71 cal ka BP). However, during the latter part of MIS 4, the sequences belonging to ASVs 5, 7 and 57 were also recorded (Figs. 4, 5 and Supplementary Fig. 2).

ASVs 5 and 10 were the dominant variants of MIS 3 (57—29 cal ka BP) (Fig. 4), whereas, ASVs 7, 38, 57, and 18 were accessory variants recorded at 37, 42, 34 and 42 cal ka BP, respectively (Fig. 5 and Supplementary Fig. 2).

MIS 2 and MIS 1 (after ~ 29 cal ka BP) were marked by having the most diverse assemblages of *N. pachyderma* ASVs (Fig. 4). The most abundantly sequenced were ASVs 5, 10 and 18 (Fig. 5). A striking change in the *N. pachyderma* genotypes was the occurrence of a large number of sequences belonging to ASV 18, which was the most abundantly sequenced ASV representing the major change in *N. pachyderma* genotypes—the replacement of single A nucleotide by a double C. Noticeably, this ASV was quite rare in the preceding stages and constituted up to 100% of the *N. pachyderma* sequences after ~ 20 cal ka BP (Figs. 4, 5). The substitution of a single A by a double C was also observed in ASVs 47 and 88, whose peaks occurred at 19 and 3.4 cal ka BP. At that time, single peaks of ASVs 16 and 57 were recorded as well (Fig. 5 and Supplementary Fig. 2).

## Discussion

Until now, molecular systematics of planktonic foraminifera focused on distinction of cryptic species, called also “genetic types” or “genotypes” that have been observed in almost every morphospecies^11^. These genetic types usually differ by a substantial number of changes (substitutions or indels) that allow to easily recognize them based on sequences of 18S barcoding gene. However, with the development of high-throughput sequencing it became obvious that some genomic variants also exist within the genetic types. Usually, these variants are clustered into so called Operational Taxonomic Units (OTUs) considered as an equivalent of species or genetic type^34^. Recently, it has been proposed to replace OTUs by the Amplicon Sequence Variants (ASVs), which correspond to the exact sequence types generated by high-throughput sequencing after filtering out spurious sequences^35^.

In our study, we used ASVs rather than OTUs because they more accurately reflect genomic variations observed in metabarcoding data. It does not exclude that some ASVs may have resulted from technical errors produced during the processing of *seda*DNA samples. Technical errors are particularly probable in the case of less abundant occurrences of ASVs characterized by single substitutions (Fig. 2 and Supplementary Fig. 1). These errors, typically introduced during the polymerase chain reaction (PCR) amplification process, are expected to be responsible for less than 1% of the divergence, which, in our case, corresponds to a single substitution. Therefore, it seems reasonable to assume that ASVs differing by more than one substitution (for example ASV 18, in which a single A nucleotide was replaced by a double C) are the result of biological variation. We also assume that ASVs that abundantly occurred in different samples would be difficult to explain by random technical errors. Therefore, further discussion will focus on the most commonly sequenced ASVs (ASVs 5, 7, 10, and 18).

It is important to mention that different ASVs can co-exist in the same specimen, as the result of intragenomic polymorphism that has been shown to be widespread in different taxonomic groups of foraminifera^12,36^ and other protists^37,38^. The intragenomic polymorphism was usually recognized as natural biological variability resulting from different rates of concerted evolution^39^, the multinucleate genomic organization^38^ or interspecific hybridization^40^. In the case of *N. pachyderma*, the intragenomic polymorphism was observed^11^, however, the level of natural intra-genomic variability was poorly studied. The public PFR2 database contains only ASV 18 of *N. pachyderma* type I, however other ASVs have been observed co-existing in the same specimens (R. Morard, *pers.commun.).*

Our study suggests that certain *N. pachyderma* ASVs occurred in relation to oceanographic changes observed at the Yermak Plateau during the last 140 cal ka BP. For each MIS, a dominant ASV could be identified (Fig. 3) and its presence correlated with environmental variables, especially the inflow of Atlantic water (AW) and associated variations in sea-ice cover and phytoplankton productivity. During the penultimate maximum glaciation the coring site was either covered by the Saalian Spitsbergen-Barents Sea-Ice sheet or an 800 to 1,300 m thick floating ice shelf extending far into Fram Strait^41,42^. Consequently, the ASVs recorded in MIS 6 are revealed from sediments that accumulated during times of intensified Atlantic Water advection that locally allowed seasonally ice-free waters. This enabled primary production is reflected in peak planktonic foraminifera shell accumulation rates during MIS 6^43^. There is a general agreement that during the last glacial maximum (MIS 2) no ice shelf but a perennially ice cover persisted over the coring site^42^. However, benthic foraminifera data from the Yermak Plateau evidence periods of enhanced AW advection and Last Glacial Maximum paleoproductivity that was significantly reduced, but was still higher than values for modern, permanently ice-covered areas^43,44^.

ASV 7 dominated the oldest part of our record (MIS 6 and MIS 5) (Figs. 3, 4). It is likely that ASV 7 was well adapted to severe environmental conditions, as this period was characterized by the presence of floating ice shelf cover and limited food supply. During the entirety of MIS 6 and MIS 5, the study site was located in the proximity of the Svalbard-Barents Ice Sheet margin, and ice shelf was present in the area^45^. The destabilization of the ice sheet and seasonal sea-ice retreat^45^, followed by peaks of paleo-productivity^46^, took place only occasionally. For instance, during MIS 5, only 2 phases of reduced sea-ice cover (associated with the release of ice-trapped organic material) were recorded at 112 and 95 cal ka BP^45^, which coincides with slight peaks in the occurrence of different *N. pachyderma* ASVs (Fig. 4). In contrast to the core site’s biomarker record^45^, previous studies have described MIS 5 (especially the Eemian interglacial period, 124–119 cal ka BP) as a period of high sea-surface temperatures^47^ reduced sea-ice conditions^48^, and increased primary production^46^ Therefore, the low abundance of biomarkers may not reflect closed sea-ice cover, but may have resulted from their removal via the grazing of sea-ice algae by primary consumers and/or degradation in the water column/sediment^49,50^. The low abundance of *N. pachyderma* sequences in the oldest sediment intervals may have also been affected by DNA degradation. This latter possibility should be confirmed by further studies and supported by other independent proxies.

Throughout the core, the presence of both ASV 5 and ASV 10 correlated with open-water conditions and increased primary production^45^ (Fig. 5). However, it is likely that ASV 5 dominated primarily during short-term, episodic sea-ice breakups, while ASV 10 required longer, open-water periods for development. ASV 10 dominated the *N. pachyderma* assemblage during early MIS 4 (71–70 cal ka BP) (Fig. 5), which was characterized by intensive AW inflow, open-water conditions, and increased phytoplankton production^43,45^. Starting in late MIS 4, ASV 5 occurred more abundantly, corresponding to a weakening of the AW inflow and enhanced sea-ice formation^45^. However, the major peaks of ASV 5 (Fig. 4) coincided with slight peaks in the concentration of phytoplankton biomarkers that was noted during MIS 3 (~ 45, 38 and 33 cal ka BP) and MIS 2 (~ 29, 27 and 24 cal ka BP)^45^. These concentrations coincide to periods of increased primary productivity triggered by enhanced inflows of warm AW and, hence, favor seasonal sea-ice retreat^45,51^. Therefore, it appears that *N. pachyderma* peaks for ASV 5 and ASV 10 may be a response to periodic amelioration of environmental conditions.

The appearance of ASV 18 in the latter part of MIS 2 and MIS 1 was the most remarkable change noted in the *N. pachyderma* assemblage (Figs. 3, 4) and coincided with the onset of deglaciation following the Last Glacial Maximum (LGM)^43^. The Svalbard Barents Ice Sheet retreat started on the Yermak Plateau around 20 cal ka BP^43^, while rapid disintegration started ~ 15 cal ka BP^53^. The retreating ice sheet reopened the pathway for the Yermak Plateau Current, which transports AW^43,51^. This may have stimulated evolution or genetic diversification. Relatively stable environmental conditions were established ~ 10 cal ka BP with increased advection of AW, seasonal sea-ice cover, and an enhanced supply of organic matter from algal blooms^43,51^.

Our results suggest that molecular analyses at finer levels can provide valuable information regarding the occurrence of different ASVs through time as well as their relations to climatic and oceanographic changes in the Arctic Ocean during the late Quaternary. The important advantage of paleo-metabarcoding is that it reveals changes over time at the population level. Assuming that the most common ASVs reported here are representative of different populations, the population structure of Arctic *N. pachyderma* has changed over the last 140 kyrs. The fluctuating relative frequencies of ASVs may be related to changes in environmental conditions and implies that they have different ecological preferences.

The fact that different ASVs have been observed in the same specimen as a result of intragenomic polymorphism does not necessarily contradicts their usefulness as paleoceanographic proxies. The intragenomic sequence variants of rRNA genes are commonly observed in multicellular organisms and assume to play a critical role in gene expression^54^. The role of these variants in foraminifera is less well understood, however, their relative frequency seems to change depending on biogeographic distribution in some benthic species^36^. For example, in the genus *Ammonia*, expansion segment polymorphism showed clear biogeographical differences, as some variants were present only in specific localities and/or proportion of observed variants varied between localities. These finding supported our assumption that the occurrence and relative abundance of intragenomic ribosomal variants may be correlated to ecological conditions.

To conclude, our study suggests that among the four common Arctic *N. pachyderma* ASVs, one (ASV 18) is clearly related to peaks in phytoplankton biomarkers, which indicate periods of reduced sea-ice cover associated with phytoplankton productivity^45^. Although this ASV was almost entirely absent in the oldest part of the record, despite the presence of the biomarkers’ peaks, this may be explained by the age of the samples and consequent DNA degradation. It is also possible that the ASV 18 was present at that time, but its abundance was too low to provide a distinct DNA trace.

The other three ASVs (ASV 5, ASV 7, and ASV 10) most likely belonged to populations with wider ecological tolerances than ASV 18, as they were also recorded during periods of less-favorable environmental conditions (e.g., the presence of perennial ice cover). However, to confirm these conclusions and to appreciate the full potential of ASVs as new proxies,it is essential to increase our knowledge concerning the molecular ecology of modern planktonic foraminifera. As the present is the key to the past, metabarcoding data on living-species distributions and their population structures are indispensable to the accurate interpretation of paleo-metabarcoding data and the use of foraminiferal genomic variants as indicators of changing environmental conditions.

## Methods

### Coring location

The Yermak Plateau is located in the vicinity of the main gateway for the AW and Polar water (PW) exchange between the Atlantic and Arctic Oceans^55^ (Fig. 1). Two major water currents regulate this water exchange: the West Spitsbergen Current (WSC) and the East Greenland Current (EGC)^56,57^. WSC transports relatively warm, saline AW northwards along the Western Spitsbergen coasts. Near the northern coasts of Spitsbergen, WSC separates into an eastern branch (North Spitsbergen Current) and western branch (Yermak Slope Current)^58^. Cold and less saline PW, along with sea ice, enters the Greenland Sea via the western Fram Strait and flows southward as the EGC along the Greenland shelf^55^ (Fig. 1).

Kastenlot core PS92/0039–2 was retrieved during the TRANSSIZ PS92 (ARK-XXIX/1) cruise of the R/V Polarstern in 2015 (Table 3) along the eastern flank of the Yermak Plateau (Fig. 5). The core was sampled onboard, and the sediments were transferred to a set of 1 m-long plastic boxes and returned to the Alfred Wegener Institute in Bremerhaven, Germany. Samples were stored at 4 °C. To perform ancient DNA analyses, the untouched sediment archives were subsampled every 5 cm. Approximately 10 g of sediment were collected with disposable spatulas and transferred to sterile containers. To avoid contamination, samples were taken from the inner part of the core. Outer sediment layers that had contact with the plastic boxes were discarded. Directly after being collected, samples were frozen at − 20 °C and shipped to the Institute of Oceanology PAN in Sopot, Poland.

**Table 3:** Location of sampling station.

Date	Station	Latitude	Longtitude	Depth [m]	Core length [cm]
11/06/2015	PS92/0039–2	81° 56.99′N	13° 49.70′E	1,464	850

### Age model and multi-proxy analysis of the core

In our study, we applied the previously published age model^45^ of the PS92/0,039–2 core, which was further validated and refined^59,60^. However, we decided to use the initial version of the age model^45^, as our results were directly compared to their paleoceanographic record. This age model was based on 8 age-fixed points inferred from (a) accelerator mass spectrometry radiocarbon dating of foraminiferal tests, (b) the correlation of carbonate content and magnetic susceptibility to the core PS1533-3^61^ and, (c) the occurrence of the benthic foraminifera *Pullenia bulloides*, which is a stratigraphic marker for the 81 ka event^62^. The age of the bottom of the core was estimated to be ~ 160 cal ka BP. Herein, we present only 140 cal ka BP, because the oldest part of the core was almost barren of *N. pachyderma* sequences.

A multi-proxy reconstruction of glacial-interglacial changes in the region^45^ were reconstructed based on sedimentary proxies (grainsize, TC/TOC, δ^13^C), phytoplankton (IP_25_, HBI III, dinosterol, brassicasterol) and terrigenous (campesterol, β-sitosterol) biomarkers, supported by magnetic susceptibility of the sediment. A detailed description of the paleoceanograpic development of the eastern Yermak Plateau during the late Quaternary is available from^45^.

### DNA analysis

Total DNA was extracted from each sample using DNeasy Power Max Soil DNA isolation kit (Qiagen, Hilden, Germany). The targeted foraminiferal DNA fragment was located in Helix 37 of ribosomal DNA (SSU rDNA), which is present in all foraminifera and appears to be specific for this group^63^. The hypervariable 37f region was amplified using forward s14F1 (5′-XXXXXCGGACACACTGAGGATTGACAG-3′) and reverse 15 s (5′-XXXXXCCTATCACATAATCATGAAAG-3′) primers tagged with a unique sequence of 5 nucleotides appended to their 5′ ends. For each sample, 5 to 10 PCR replicates were prepared. The amplicons were quantified using a Qubit 3.0 fluorometer (Thermo-Fisher Scientific Inc., Waltham, MA, USA) and pooled in equimolar quantities. The pool was purified with High Pure PCR Cleanup Micro Kit (Roche Diagnostics GmbH, Mannheim, Germany). The sequence library was prepared using the Illumina TruSeq library-preparation kit (Illumina Inc., San Diego, CA, USA) and loaded onto a MiSeq instrument for a paired-end run of 2*150 cycles. The post-sequencing data processing was performed using the SLIM pipeline^64^ and included sample demultiplexing, assembled into full-length sequences, and chimera filtering. Sequences were clustered into ASVs^35^ with 100% similarity and assigned using a foraminifera nucleotide database. The results were presented as an ASV-to-sample table. Only ASVs comprising more than 1,000 reads and samples comprising more than 100 reads were kept for further analyses. To find different genotypes in the 37f hypervariable region, the sequences were combined and manually analyzed using Seaview^65^. DNA secondary structures were constructed using mfold^66^ using default parameters. The abundance of ASVs assigned to *N. pachyderma* was expressed as the percentage (%) of foraminiferal sequences.

## Supplementary information

Supplementary Figures

## Data Availability

The data set is stored in the Oceanographic Data and Information Management System of the Institute of oceanology Polish Academy of Sciences. The data can be accessed at https://www.iopan.pl/Paleo/research.html (file name: PS92-039-2_Neogloboquadrina_pachyderma).
