# Supplementary material for: Planktonic foraminifera genomic variations reflect paleoceanographic changes in the Arctic: evidence from sedimentary ancient DNA
Source: Sci Rep. 2020 Sep 15;10:15102. doi: 10.1038/s41598-020-72146-9 (PMC7492196; doi:10.1038/s41598-020-72146-9)
Supplement: Supplementary file 1 — Supplementary Figures [file 41598_2020_72146_MOESM1_ESM.pdf]

Planktonic foraminifera genomic variations reflect paleoceanographic changes in the Arctic: evidence from sedimentary ancient DNA

Joanna Pawłowska[1*], Jutta Wollenburg[2], Marek Zajączkowski[1], Jan Pawlowski[1,3]

[1]Institute of Oceanology Polish Academy of Sciences, Sopot, Poland

[2]Alfred Wegener Institute, Bremerhaven, Germany

[3]University of Geneva, Geneva, Switzerland

*Corresponding author, email: pawlowska@iopan.pl

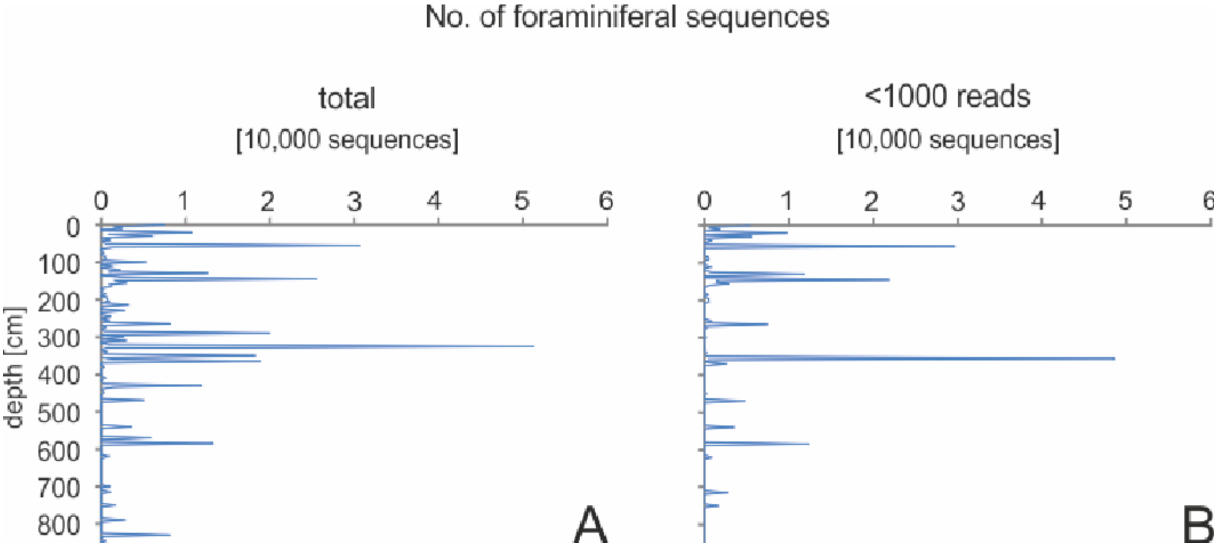

Supplementary Fig. 1. Number of benthic and planktonic foraminifera sequence reads found in the dataset. Total number of sequences (A) and sequences belonging to ASVs that comprised more than 1000 reads (B) are presented.

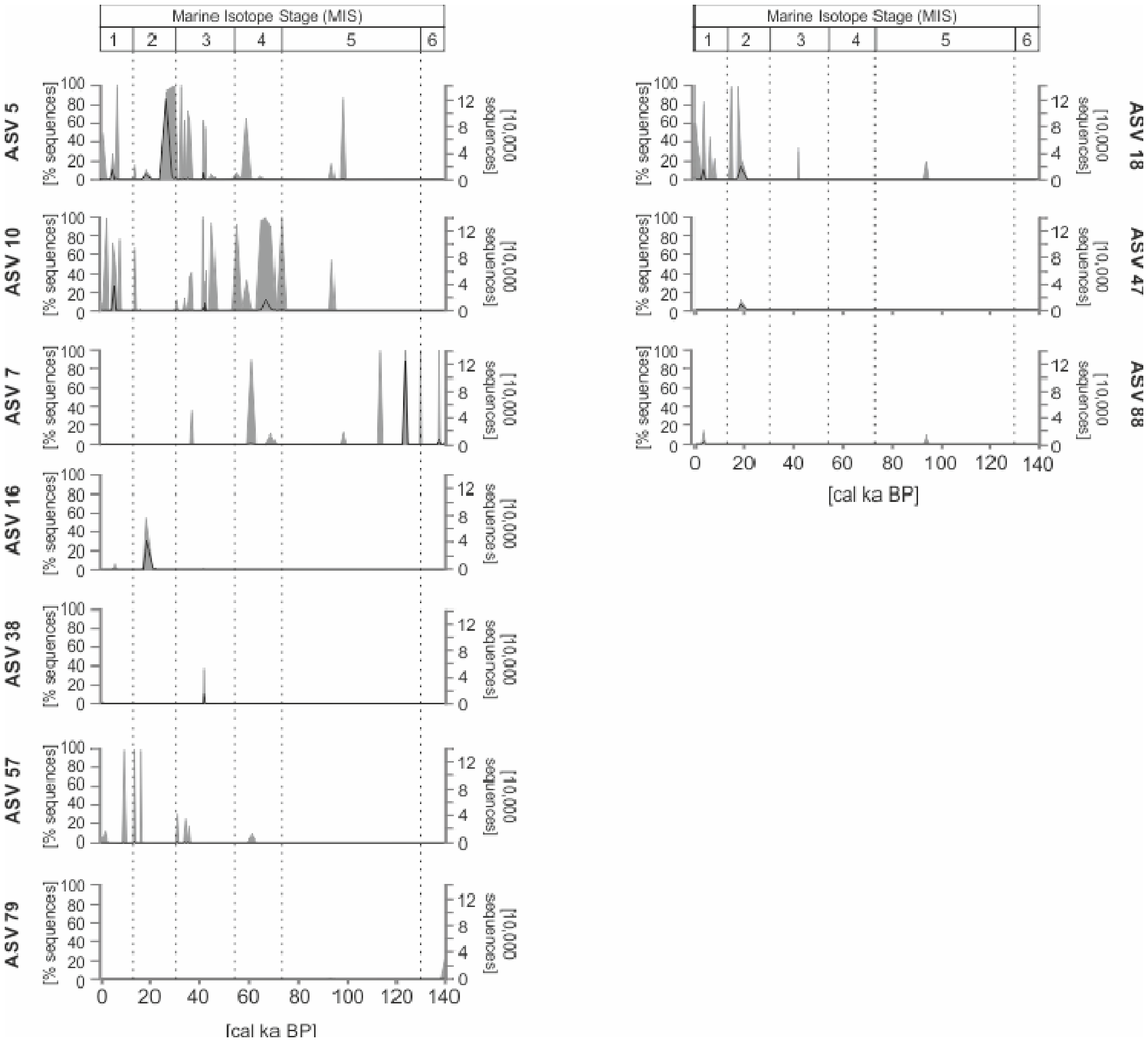

Supplementary Fig. 2. The abundance of the ASVs that constituted more than 1% of *Neogloboquadrina pachyderma* sequences, expressed as a percentage of *N. pachyderma* sequences (grey shading) and number of sequence reads (black line). ASVs in which a “double C” substitution was observed are presented on the right panel, while “single A” ASVs are presented on the left panel. The ASVs are ordered from the most to the least abundantly sequenced.
